# Separating homeologs by phasing in the tetraploid wheat transcriptome

**DOI:** 10.1186/gb-2013-14-6-r66

**Published:** 2013-06-25

**Authors:** Ksenia V Krasileva, Vince Buffalo, Paul Bailey, Stephen Pearce, Sarah Ayling, Facundo Tabbita, Marcelo Soria, Shichen Wang, IWGS Consortium, Eduard Akhunov, Cristobal Uauy, Jorge Dubcovsky

**Affiliations:** 1 Dept. Plant Sciences, University of California, Davis, CA 9561, USA; 2 The Genome Analysis Centre, Norwich Research Park, Norwich NR4 7UH, UK; 3 Microbiology, University of Buenos Aires, INBA-CONICET, Buenos Aires, Argentina; 4 Department of Plant Pathology, Kansas State University, Manhattan, KS 66506, USA; 5 International Wheat Genome Sequencing Consortium; 6 John Innes Centre, Norwich Research Park, Norwich NR4 7UH, UK; 7 Howard Hughes Medical Institute, Chevy Chase, MD 20815, USA

**Keywords:** Transcriptome assembly, multiple k-mer assembly, wheat, polyploid, *Triticum urartu*, *Triticum turgidum*, pseudogenes, phasing, gene prediction

## Abstract

**Background:**

The high level of identity among duplicated homoeologous genomes in tetraploid pasta wheat presents substantial challenges for *de novo *transcriptome assembly. To solve this problem, we develop a specialized bioinformatics workflow that optimizes transcriptome assembly and separation of merged homoeologs. To evaluate our strategy, we sequence and assemble the transcriptome of one of the diploid ancestors of pasta wheat, and compare both assemblies with a benchmark set of 13,472 full-length, non-redundant bread wheat cDNAs.

**Results:**

A total of 489 million 100 bp paired-end reads from tetraploid wheat assemble in 140,118 contigs, including 96% of the benchmark cDNAs. We used a comparative genomics approach to annotate 66,633 open reading frames. The multiple k-mer assembly strategy increases the proportion of cDNAs assembled full-length in a single contig by 22% relative to the best single k-mer size. Homoeologs are separated using a post-assembly pipeline that includes polymorphism identification, phasing of SNPs, read sorting, and re-assembly of phased reads. Using a reference set of genes, we determine that 98.7% of SNPs analyzed are correctly separated by phasing.

**Conclusions:**

Our study shows that *de novo *transcriptome assembly of tetraploid wheat benefit from multiple k-mer assembly strategies more than diploid wheat. Our results also demonstrate that phasing approaches originally designed for heterozygous diploid organisms can be used to separate the close homoeologous genomes of tetraploid wheat. The predicted tetraploid wheat proteome and gene models provide a valuable tool for the wheat research community and for those interested in comparative genomic studies.

## Background

Whole genome duplication events, or polyploidization, have occurred repeatedly throughout the evolutionary history of flowering plants[1,2]. Many currently cultivated species are recent polyploids, formed through either inter-specific hybridization (allopolyploids, such as wheat, oats, canola, peanut, and cotton)or intra-specific hybridization (autopolyploids, such as apple, strawberry, watermelon, and alfalfa)[2]. In addition,homoeologs in older polyploid species, such as maize (11-15 million years since polyploidization)[3]have had a longer time to diverge through deletions, loss of function, neo-functionalization, and sub-functionalization processes (usually referred to as diploidization). These processes confer polyploid species an increased evolutionary plasticity, whichpromotes speciation and adaptation to new environmentsand contributes to the huge success of polyploidy in plant evolution[2,4].When diploidization processes continue over long periods of time, they lead to the formation of paleo-polyploid species (for example, rice), which are difficult to differentiate from true diploid species. However, genomic studies haveprovided convincing evidence of ancient whole genome duplication events in the early monocot and dicot lineages suggesting that polyploidy was part of the evolution of most current angiosperms [5,6].

Wheat (*Triticum *spp.) was domesticated at the dawn of agriculture approximately 10,000 years ago and has since been adapted to grow in a broad range of climates throughout the world [4]. Most cultivated wheat varieties belong to two species; tetraploid *Triticum turgidum *L. (durum or pasta wheat, genomes AABB) and hexaploid *T. aestivum *L. (common wheat, genomes AABBDD). The tetraploid wheat genome originated from an inter-specific hybridization event occurring less than 0.5 million years ago, which combined the AA genome of *T. urartu *Tumanian ex Gandilyan and the BB genome of an unknown grass species related to *Aegilops speltoides *Tausch[7-9].Common wheat, *T. aestivum *, evolved from a second round of inter-specific hybridization and genome duplication that occurred shortly after domestication and combinedthe tetraploid AABB genomes of cultivated *T. turgidum *and the DD genome of the wild grass *Aegilops tauschii *(Coss.) Schmalh[4].

The diploid progenitors of polyploid wheat species diverged from a common ancestor only 2.5-4.5 million years ago[10], which is reflected in a high average identity (approximately 97%) among coding regions of different wheat homoeologs. However, this average varies greatly among gene classes that are subject to different evolutionary pressures [11]. For example, conversion events (unequal crossing-over between tandemly-duplicated paralogs) and diversifying selection processes are known to accelerate the divergence rate between members of the disease resistance gene family [12-14].

Wheat intergenic regions diverge even faster than rapidly evolving gene families due to high levels of methylation and increased rates of insertions and deletions, which are associated with the abundance of repetitive elements in these regions [15]. These rapid changes in the intergenic regions can affect neighboring genes and result in rapid rates of gene insertion, deletion, and transposition[16].The potentially negative effects associated with gene deletions are buffered by polyploidy[17-20].Transposition of genes and gene fragments by adjacent retroelements results in higher proliferation of pseudogenes in the large polyploid *Triticeae *genomes compared to other grass species with smaller genomes[19,21].In addition, increased divergence of alternative splicing variants between the diploid progenitors further diversifieshomoeologs'gene structure (and potentially their function) in the polyploid wheat species[21]. The dynamic nature of these large genomes needs to be considered in the development of strategies to characterize the wheat gene complement.

In species with large genomes, *de novo *transcriptome assemblies are an effective strategy to access the gene spacewhile avoiding the highly repetitive intergenic regions. In wheat, for example, the transcribed gene-coding regionsrepresent only 1% to 2% percent of the totalgenome[22]. Rapid growth in throughput, quality, and accessibility of next-generation sequencing technologies, together with improvements in *de novo *transcriptome assembly algorithms have fostered a multitude of transcriptome sequencing projects. With increased access to next generation sequencing, many plant *de novo *transcriptome assemblies have been published and several different assembly algorithms have been proposed[23-25]. However, the challenges specific to *de novo *transcriptome assembly of a young polyploid speciessuch as tetraploid wheat are just starting to be addressed[26,27]. Particularly important is the correct separation of close homoeologs, since there are known examples of different homoeologs contributing differently to important agronomic traits (for example, wheat *VRN1 *homoeologs[28]). Correct separation of homoeologs is also important for breeding applications, marker development, and downstream genomics analyses.

Three recent studiesof hexaploid wheat transcriptomes[27,29,30] highlight the difficulties of assembling closely related homoeologs in a polyploid species. Schreiber *et al*. (2012) observed that most homoeologs were collapsed into chimeric contigs when hexaploid wheat transcriptomes were assembled using either Velvet/Oases (60% to 80% chimeric sequences) or Trinity (50% chimeric sequences). A computationally-intensive two-stage assembly using the MIRA assembler helped to reduce the number of chimeric homoeolog sequences to 18%, thus partially solving the polyploid problem at the assembly step [27].An alternative strategy was used by The International Wheat Genome Sequencing Consortium (IWGSC): genome-specific contigs of hexaploid wheatwere generated by sorting individual chromosome arms usingflow cytometry and sequencing and assembling each of them separately[21,31,32].

In this paper, we present abioinformatics pipeline that addresses the challenges of *de novo *transcriptome assembly of the closely related genomes of tetraploid wheat. Using this pipeline, weassembled, annotated and analyzedthe transcriptome of *T. turgidum *cv. Kronos and of its closest diploid relative *T. urartu*.This diploid wheat transcriptome together with a reference dataset of 13,472 full-lengthwheat cDNAs were used to evaluate the effect of different parameters on the quality of the tetraploid wheat assembly.We developed post-assembly processing strategies and software that allowed us to generate homoeolog-specific sub-assemblies. Finally, we used comparative genomics approaches to annotate open reading frames and predicted proteins, predict pseudogenes and artificially fused transcripts, and generate gene models to increase the value of this resource.

## Results and discussion

### Sequencing and evaluation of experimental and digital normalization

In total, we sequenced 248.5 million and 488.9 million paired-endIllumina reads (100 bp each) for *T. urartu *and *T. turgidum *cv. Kronos, respectively (Additional File 2, Table S1).The raw reads were submitted to the Short Read Archive (SRA) and linked to their respective NCBI BioProjects PRJNA191053(*T. urartu*) and PRJNA191054(*T. turgidum*). After trimming Illumina adapter sequences with Scythe and poor quality bases with Sickle (see Materials andmethods) the average read length was94 bp for *T. urartu *and 96 bp for *T. turgidum*. The number of reads obtained from individual RNA-seq libraries varied from 20.3 to 137.1 million reads and is summarized in Additional file 2, Table S1.

#### Double-stranded DNA nuclease (DSN) normalization

Results from DSN are described in Figure S1 (Additional file 3). First, we evaluated the fold change in abundance of four marker genes by quantitative RT-PCR (Additional file 3, Figure S1A). Rubisco, one of the most highly expressed genes, showed an 11- to 13-fold decrease in transcript levels after normalization, whereas transcripts of a low abundance NBS-LRR geneshowed a slight increase after normalization (Additional file 3, Figure S1A). We then evaluated the relative abundance of Illumina reads mapped to a reference set of full-length wheat cDNA transcripts and additional high abundance geneswith and without normalization (Additional file 3, Figure S1B,C). Our results showed that DSN normalization resulted in an enrichment of the low abundance transcripts and a reduction of the most abundant transcripts relative to the control without DSN normalization. There were a substantial number oftranscripts detected only after normalization (new points to the left of the red reference line in Figure S1C), which indicates that our DSN normalization contributed to a more comprehensive transcriptome assembly.

#### Digital normalization

In addition to the experimental DSN normalization and prior to assembly, we performed a digital normalization of the reads using the khmerprogram [33](see Materials and methods). This normalization is designed to reduce redundancy in the RNA-seq data and accelerate assembly. We tested the effect of digital normalization on assembly quality using a previously published RNA-seq library of *T. turgidum *cv. Langdon [34]. The 28 million reads present in this library were reduced to 9 million reads after digital normalization. Both sets of reads were assembled using our multiple k-mer size assembly pipeline (see next section) and the resulting contigs were aligned to the 13,472 full-length wheat cDNA benchmark set [35]using BLASTN (E-value 1e^-20^, >90% identity). Additional file 4, Figure S2 shows thatboth datasets have identical distributions of the number of reference genes assembled at different levels of coverage (correlation between distributions *R *= 0.99989). This result confirmed that digital normalization had no significant negative effects on the quality of assembliesgenerated by our multiple k-mer length assembly pipeline. Digital normalization reduced the number of paired-end reads five-fold (Table 1), thus greatly reducingthe time and resources required for the multiple k-merassemblies.

**Table 1:** The *T. urartu *and *T. turgidum *final assembly statistics

	*T.urartu*	*T.turgidum*
100-bp paired-end reads (*n*)	248.5 million	488.9 million
Reads after digital normalization^a ^(*n*)	47.3 million	110.7 million
Contigs (*n*)	86,247	140,118
Mean contig size (bp)	1,417 bp	1,299 bp
Min contig size (bp)	212 bp	298 bp
Max contig size (bp)	17,959 bp	26,226 bp
GC content (%)	49%	49%
Total transcriptome size (Mb)	122 Mb	181 Mb
Reads mapping to the assembly (% of total reads)	82.2%	81.5%
Reads mapped in proper pairs (% of total reads)	73.0%	71.5%
Unique alignments (% of total mapped)	52.8%	76.7%
Benchmark genes^b ^assembled > 50% length in a single contig	12,693 (94%)	12,961 (96%)
Benchmark genes^b ^assembled > 90% length in a single contig	10,727 (80%)	10,197 (76%)

^a^Elimination of *Homo sapiens,Escherichia coli*, wheat mitochondrial, rRNA, and chloroplast sequences resulted in the elimination of 0.5% of the digitally normalized reads in *T. urartu *and 0.6% in *T. turgidum*.

^b^13,472 full-length cDNAs from the RIKEN Plant Science Center Japan [35].

### Distribution of percent identity and SNP distances between A and B homoeologs

**Figure 1:**
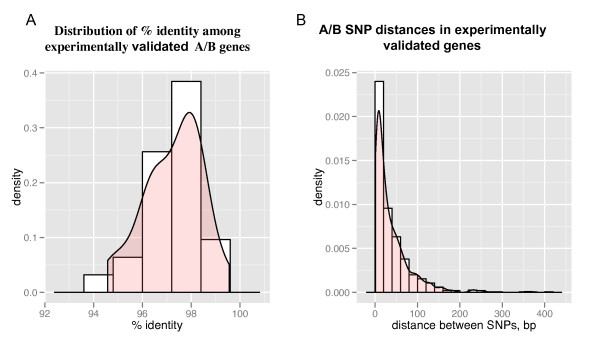
**Divergence of A and B transcripts**. (**A**) Distribution of percent identity between A/B homoeologous genes in a set of 26 experimentally validated genes (52 homoeologs). Mean = 97.3%; SD = 1.20%. (**B**) Distribution of distances between 707 single nucleotide polymorphisms (SNPs) between homoeologs in tetraploid wheat coding regions. Mean = 37.8 bp; SD = 47.1 bp; Median = 27 bp.

Several of the programs used in our assembly pipeline require input parameters that are dependent on the level of divergence between the homoeologousgenomes and/oron the average distance between single nucleotide polymorphisms (SNPs). To estimate these two parameters we analyzed the coding sequences of 52genes (26 A/B genome homoeolog pairs, average size 1,199 bp, Supplemental dataset 1[36]), which were previously sequenced and annotated in our laboratories. DNA sequence identity (excluding gaps) between A and B coding regions showed a normal distribution (Shapiro-Wilk test *P *= 0.40)with a mean of 97.26% and a standard deviation of 1.20%(Figure 1A). Based on this result,we estimated that a minimum threshold of 94%identity (≤12 SNPs per 100 bp paired-end fragments) would include approximately 99% of all identity values between true homoeologs and allow roughly 99% of the paired-end reads to map to both homoeologs. With a minimum threshold of 95% identity (≤10 SNPs per 100 bp paired-end fragments) the previous proportions were reduced to 95% of the homoeologs and mapped reads.

Poisson processes for SNPs imply exponential distributions of inter-SNP distances and hence long tails [37].The frequency of inter-SNP distances found in this study between wheat homoeolog coding regions also decreased exponentially with inter-SNP distance (Figure 1B). For this set of 52 genes, the mean distance between adjacent SNPs was 37.8 bp (standard deviation of 47.1 bp), which is close to the average distance of 32.9 bp estimated from the 97.26% percent identity and the 1,199 bp average lengthof the 26 manually-curated homoeolog pairs used in our dataset (Figure 1B).

**Figure 2:**
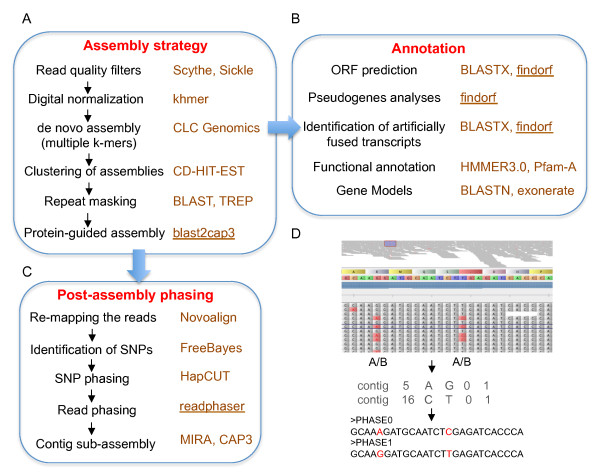
**Strategies for genome-specific assembly and annotation of the tetraploid wheat transcriptome**. (**A**) Overall assembly pipeline. Functional steps are listed on the left and specific programs used for each step on the right. Programs developed during the course of this study are underlined. (**B**) Steps used in the annotation. (**C**) Post-assembly processing pipeline using phasing to separate homoeolog-specific sequences. (**D**) Illustration of the phasing process. Reads are re-aligned to the reference transcriptome, single nucleotide polymorphisms (SNPs) between homoeologs are identified (in red), and phased. The example shows the phasing of A and C SNPs at positions 5 and 16 in phase 0 and G and T SNPs in phase 1.

This level of polymorphism, the variable distances between adjacent SNPs, and the need to separate close homoeologspose challenges to most transcriptome assemblers, which were designed and tested for lower levels of intraspecific heterozygosity and were not required to separate close haplotypes. To address this problem we applied several post-assembly processing tools that integrateavailable and novel software to generate homoeolog-specific sub-assemblies.The overall assembly strategy is described in Figure 2A, the annotation procedures in Figure 2B, and the specific steps to separate the collapsed homoeologs into homoeolog-specific sequences are illustrated in Figure 2C. A detailed description of each of the different steps is included below.

### Effect of different k-mersizeson the assembly of diploid and tetraploid wheat transcriptomes

**Figure 3:**
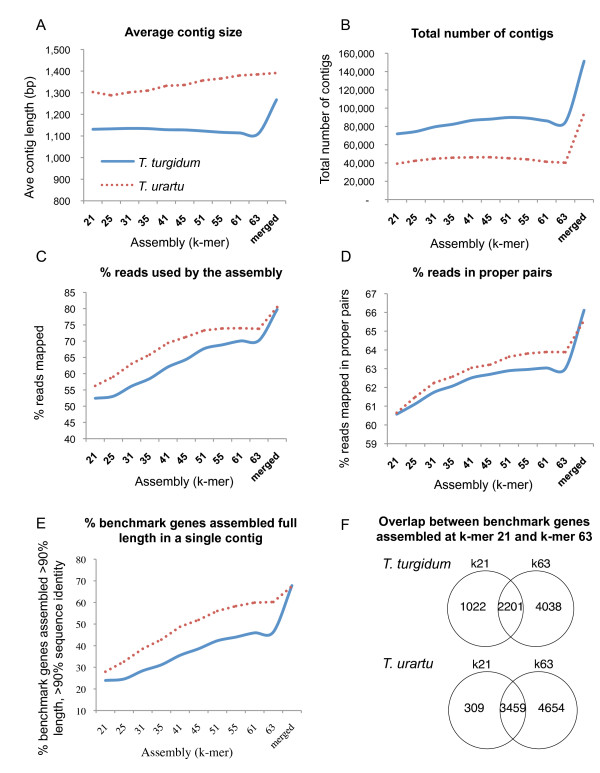
**Comparison of the effect of different k-mers on transcriptome assembly metrics in diploid and tetraploid wheat**. *T. urartu *values are indicated by the red dotted lineand *T. turgidum *by theblue solid line. (**A**) Average contigs length. (**B**) Total number of contigs. (**C**) Percent of total reads mapped back to the assembly. (**D**) Percent of total reads that are mapped in proper pairs. (**E**) Fraction of 13,472 full-length benchmark wheat cDNAs that are assembled in a single contig. (**F**) Venn diagram showing the number of benchmark cDNAs assembled full-length (>90%) at k-mer sizes 21 and 63.

For initial reconstruction of the wheat transcriptome we useda de Bruijn graph *de novo *assembly algorithm implemented in CLC Genomics v5.5. Since the word size (or k-mer size) is one of the key parameters in constructing de Bruijn graphs, we evaluated the effect of 10 different k-mer sizes(ranging from 21 to 63, the maximum permitted in CLC) on the assembly of tetraploid and diploid wheat transcriptomes.At each k-mer size, we assessed basic assembly metrics, includingthe total number of contigs, average contig size and the proportion of reads assembled.In addition to these basic measures, we estimated completeness of our assemblyby assessing the proportion of13,472 benchmark cDNA sequences[35]assembled at full length in a single contig (Figure 3A-E, Additional file 2, Table S2).

While the resulting number of contigs and average contig size differed very little across the range of k-mer values (Figure 3A, B, Additional file 2 Table S2), all other metrics indicated an improvement in assembly quality with increased k-mersize up to k-mer 61 (Figures 3C, D, and 3E, Additional file 2 Table S2). For both *T. urartu *and *T. turgidum*, assembly completeness increased with k-mer length; at k-mer 63, 74% and 70% of all reads utilized in the assembly compared to only 56% and 52% at k-mer 21, respectively (Figure 3C, Additional file 2 Table S2).The percent of reads mapping in proper pairs, an indicator of assembly continuity also improved with increasing k-mer size, but the gain was more modest, ranging from 61% at k-mer 21 to 63% to 64% at k-mer 63 (Figure 3D, Additional file 2 Table S2).

Figure 3E shows that a larger proportion of the 13,472benchmark cDNA sequences [35]are assembled at full length (>90% coverage) at larger k-mersizes. This metric showed clear differences between the diploid and the tetraploid assemblies for all k-mersizes,with the *T. turgidum *assemblies showing a lower proportion of fully assembled genes than the *T. urartu *assemblies. In *T. turgidum*, only 46% of benchmark genes were assembled at fulllength in a single contig at k-mer 63, while in *T. urartu*, this number was close to 60% (Figure 3E, F, Additional file 2 Table S2). This result suggeststhat *de novo *transcriptomeassemblies can be more fractionated in polyploid species with recently duplicated genomes than in their donor diploid species.

**Figure 4:**
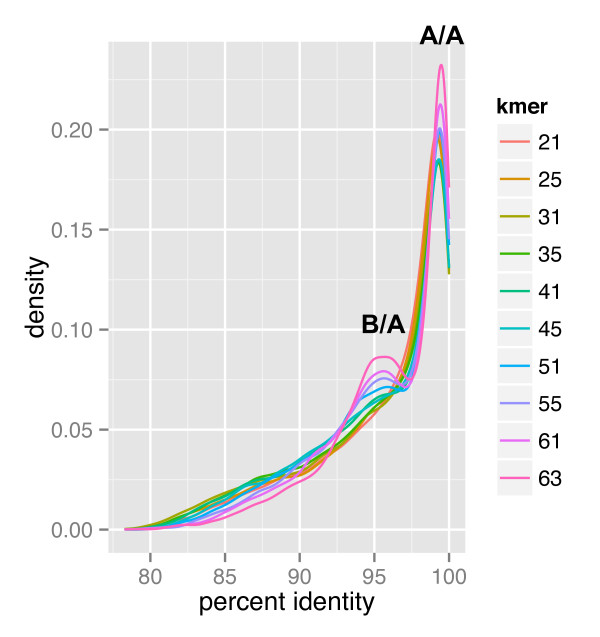
**Distribution of percent identities between *T. urartu *and *T. turgidum *merged assemblies**. The graph represents the distribution of percent identity between *T. turgidum *and *T. urartu *merged assemblies as calculated by BLASTN(E-value cutoff 1e^-20^). Densities are colored by the k-mer which contributed each contig to the merged assembly.

*Triticum turgidum *contigs that are separated correctly into A and B homoeologs should show a bimodal distribution of percent identities when compared with *T. urartu*. In Figure 4, we plotted the distribution of percent identities between the best BLAST hits between *T. urartu *and *T. turgidum *contigs, colored according to the specific k-mer assembly that contributed that contig. All k-mer sizes show a sharp peak at 99% identity that corresponds to the tetraploid A genome contigs aligned with the diploid A genome progenitor, but only the larger k-mersizes show a second peak around 96% identity (Figure 4). We interpret this result as evidence of a better separation of A and B genome contigs derived from the larger k-mersizes. Chimeric A/B assemblies are more abundant at lower k-mersizesand their intermediate percent identity values 'fill' the valley between the A/A and A/B peaks resulting in curves with a single peak (Figure 4). For all k-mersizes, identity values <94% include the most divergent 1% of the homoeologs, but most likely also include many paralogous alignments.

### Advantages and disadvantages of merged multiple k-mer assemblies

Since different k-mer sizes lead to full-length assemblies ofdifferent sets of genes (Figure 1F), we combined the contigs from the 10 different k-mer assemblies, and eliminated redundancy using the CD-HIT program (see Materials and methods), which was recently shown to produce more inclusive sets of transcripts compared with Oases and V-MATCH [38].

The advantages of this approach were evident in the improvement of several assembly metrics in the CD-HIT merged k-mer assemblies relative to the single k-mer assemblies. The CD-HIT merged k-mer datasets showed a higher fraction of reads mapping back to the assembly (Figure 3C), a higher fraction of reads mapped in proper pairs (Figure 3D), and an increase in the proportion of contigs including complete benchmark transcripts (Figure 3E) in both *T. turgidum *and *T. urartu*. However, gains from the merged dataset relative to the best individual k-mer size were greater in *T. turgidum *than in *T. urartu*. This difference was particularly clear for the last metric, where the percent of cDNAs assembled full-length in a single contig rose from 60% to 68% for *T. urartu *and from46% to 68% for *T. turgidum *(Figure 3E). It is interesting to point out that, based on our different metrics,the quality of our diploid and tetraploid transcriptomes are similar only in the merged k-mer assemblies. This result suggests that by merging assemblies from a wide range of k-mersizes it is possible to achieve similar quality for both diploid and tetraploid *de novo *transcriptome assemblies. The importance of using a wide range of k-merlengthsin the assembly of transcriptomes from polyploid species is further illustrated in Figure 3F, which shows that the proportion of genes assembled at full length at k-mer 21 but not at k-mer 63 was larger in *T. turgidum *(14%) than in *T. urartu *(4%, Figure 3F).

One disadvantage of using a multiple k-mer approach is the high redundancy generated by duplicated genes and different A/B chimeric forms of the same gene assembled at different k-mersizes. Fortunately, a large proportion of this redundancy can be eliminated using CD-HIT with the appropriate percent identity threshold. By adjusting this parameter to 95%in *T. turgidum *and 99% in *T. urartu *we reduced the initial number of contigs 4.6-fold in *T. urartu *and 5.5-fold in *T. turgidum *(Additional file 2, Table S2). A 95%identity threshold was selected for tetraploid wheat to merge most (approximately 95%) duplicated chimeric contigs (Figure 1A).This thresholdalso eliminated one member of fully-overlapping homoeolog-pairs even if they were not A/B chimeras. Many of the eliminated homoeologs were recoveredat a later stage during the phasing and reassembly of phased reads (Figure 2C). As mapping quality decreases significantly when reads map to multiple locations, the elimination of one of the members of close homoeolog pairs has the additional benefit of improving read mapping quality which is important for SNP calling and phasing in the next steps of our pipeline.

A limitation of the CD-HIT program is that it does not merge partially overlapping contigs, so additional steps were needed to combine overlapping contigs from different k-mer assemblies. To reconstructtranscripts split between partially overlapping contigs we implemented blast2cap3[39],a protein-guided assembly approach,to reconstruct partially overlapping contigs assembled at different k-mersizes (see Materials and methods, Additional file 5). Briefly, blast2cap3first clusters contigs based on similarity to a common protein and then passes each cluster to the overlap-based assembly program CAP3[40]. By operating on small subsets of contigs that have been pre-filtered using biologically-relevant information, blast2cap3 generates less artificially fused sequences as compared to assembling the entire dataset with CAP3. For this study we used seven plant protein databases (six grass species and Arabidopsis, see Additional file 2, Table S3) and a high stringency criteria (>99% identity for >100 bp, to reduce the generation of chimeric A/B clones). To further lower the risk of merging incorrect contigs based on common repetitive elements we masked all sequences using the *Triticeae *Repeat Sequence Database (TREP) (BLASTN and BLASTX, E-value cutoff 1e^-10^) before running blast2cap3. The implementation of blast2cap3 reduced the total number of contigs by 8% to 9%, reducing assembly redundancy and/or fractionation.

After these merges,the final transcriptomes included 86,247 contigs for *T. urartu *(average 1,417 bp, Supplemental dataset 2[36]) and 140,118 contigs for *T. turgidum *(average 1,299 bp, Supplemental dataset 3[36]) (Table 1). The *T. turgidum *transcriptome included 96% of the 13,472 benchmark full-length cDNA sequences [35]with a coverage >50% in single contigs and 80% with a coverage >90% (compared to 68% before blast2cap3). The *T. urartu *transcriptome showed similar parameters (94% with coverage >50% and 76% with coverage >90%, Table 1).These results suggest that our transcriptomes include a large proportion of all wheat genes. Final *T. urartu *and *T. turgidum *assemblies were filtered according to the guidelines of Transcriptome Shotgun Assembly (TSA) and deposited under TSA accessions GAKL00000000 and GAKM00000000, respectively.

After the assemblies were completed, the bioinformatics pipeline branched in two directions: one focused on the annotation of the contigs (Figure 2B) and the other aimed at separating sequences from the A and B genomes by phasing (Figure 2C). These post-assembly processes are described in detail below.

### Open reading frame prediction and functional annotation of wheat transcriptomes

The ORF prediction process was based on a comparative genomics approach implemented in the findorf program (Additional file 6) [41]. This approach relies on BLASTX alignments between transcripts and proteomes from other plant species (Additional file 2, Table S3) and Hidden Markov Model (HMM)-based Pfam domain predictions (see Materials and methods for specific parameters). In total, we predicted 76,570 ORFs for *T. turgidum *(and 43,014 for *T. urartu*, Table 2).Functional annotation of the predicted proteins using HMM-based searches against Pfam[42] (see Materials and methods) showed that the three most prominent domains in our wheat transcriptomeswereprotein kinase (Pkinase), leucine-rich repeat (LRR), and nucleotide-binding site (NBS) domains - signature domains of receptor-like kinases and plant disease resistance genes.

**Table 2:** Open reading frame prediction^a^

	*T. turgidum*	*T. urartu*
Contigs (*n*)	140,118	86,247
Non-wheat sequences^b ^(eliminated) (*n*)	558	518

*Wheat protein coding sequences*		
BLASTX, E-value cutoff 1e^-3^	96,244	59,439
Contigs with a Pfam domain (1e^-3^)	59,917	39,965
Contig sequences without BLASTX (1e^-3^) or Pfam (1e^-3^)	42,999	26,070

*Predicted open reading frames*		
Predicted ORFs (non-redundant, >30 amino acids)	76,570	43,014
Fulllength	32,548	22,868
Missing 5' end	26,723	12,225
Missing 3' end	12,792	5,376
Missing 5' and 3' end	4,507	2,545
Putative pseudogenes (frameshift and/or premature stop codon)	9,937	5,208

*Putative fused transcripts*		
Contigs with BLASTX on inconsistent strand	4,376	3,628
Contigs with >1 predicted ORFs (>30 amino acids, no repetitive elements, not a pseudogene)	2,164	1,349

Putative fused transcripts (excluding overlaps) (*n*)	6,409	4,866

^a^Open reading frames were predicted with a comparative genomics approach using the findorfprogram and BLASTX alignments (E-value cutoff 1e^-5^) between contigs and proteomes of barley, *Brachypodium*, rice, maize, sorghum, and Arabidopsis.

^b^Non-wheat sequences were identified based on taxonomic distribution of top 10 BLASTX hits against nr.

Roughly 30% of the contigs (26,070for *T.urartu *and 42,999for *T.turgidum*) did not show significant similarity to any plant protein by BLASTX (E-value 1e^-3^),nor to any Pfam domain(E-value 1e^-3^) (Table 2). These contigs are likely to include:(1) wheat-specific genes and rapidly evolving gene families;(2) expressed pseudogenes that have accumulated too many mutations;(3) non-coding transcribed sequences;(4) pieces of 5' and 3' UTRs;and (5) general assembly artifacts. Although at this point it is hard to differentiate between these possibilities, it is interesting to note that many well-studied transcriptomes, such as mouse and human, contain a substantial number of long non-protein coding RNAs (lncRNAs) [43,44]. LncRNAs have been shown to regulate a variety of cellular processes and several show increased expression in response to stress and pathogen attack in wheat [45]. The human ENCODE project has demonstrated the value of documenting and storing these non-coding sequences[46].

#### Pseudogenes

**Figure 5:**
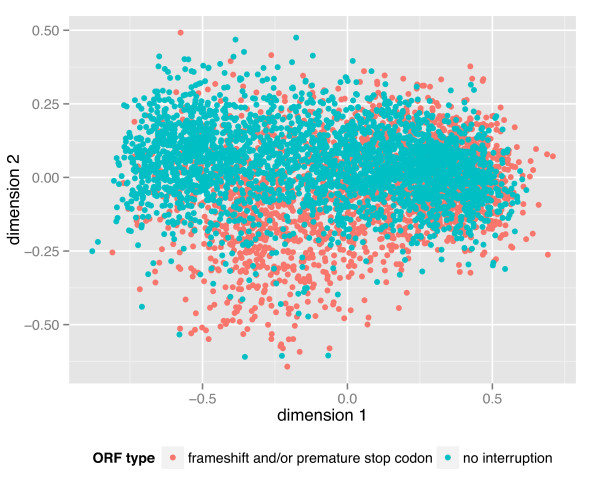
**Comparison of codon usage in predicted genes and pseudogenes**. A multidimension scaling scatterplot was generated from a random set of 3,000 full-length and 3,000 pseudogene-containing contigs. Pseudogenes were predicted by findorf by the presence of internal frameshifts or stop codon compared with known plant proteins.

Using the findorfprogram(Additional file 6)[41]we identified 5,208 ORFs in *T. urartu*(12.1%) and 9,937 in *T. turgidum*(13.0%) that were disrupted by frameshifts or stop codons (Table 2). Even though the percentages of predicted pseudogenes in these two datasets are relatively close, they are significantly different (*P* <0.0001, Fisher's Exact Test) due to the large sample size. A slightly higher proportion of pseudogenes in *T. turgidum *than in *T. urartu *is to be expected since gene duplications are known to lead to relaxed selection [47]. To validate the pseudogene predictions we compared theircodon usage with that of predicted functional genes.Pseudogenecodon usage is expected to drift towards that of intergenic DNA regions due to a lack of purifying selection [48]. Figure 5shows a multidimensional scaling plot of the distances between contigs based on the frequencies of codon usage in ORFs. The partial separation across the two-dimensional space indicates a tendency towards differential codon usage between functional and non-functional ORFs with predicted frameshift mutations or premature stop codons, which provides an independent validation for the pseudogene prediction pipeline. A partial overlap between these two classes is expected for pseudogenes of recent origin.

It is interesting to note that our estimates of the proportion of pseudogenes present in *T. urartu *and *T. turgidum*transcriptomes are lower than the 28%^a^estimate obtained from a *T. aestivum *transcriptome assembled from Roche 454 reads[21].This discrepancy is not likely to be caused by differences in pseudogene identification methods, since our findorf prediction pipeline estimated a very similar proportion of pseudogenes (27% of the ORFs) in the recently published transcriptome of *T. aestivum *variety Kukri assembled using a combination of Roche-454 and Illumina GAIIx paired-end reads[27].The higher proportion of pseudogenes observed in *T. aestivum *than in *T. turgidum *transcriptomes is unexpected given the short evolutionary time since the origin of *T. aestivum *from *T. turgidum*. It is possible that differences in sequencing technologies and/or assembly methods may have also contributed to these differences. For example, homopolymer sequencing errors in Roche 454 sequences generate frameshift mutations, which can result in an overestimation of the proportion of pseudogenes.

#### Artificially fused transcripts

During the initial ORF prediction we determined which contigs were aligned to more than one plant protein in the opposite orientation (one BLAST hit to the positive strand, and another to the negative strand). We initially identified a total of 3,628 contigs with inconsistent strands in *T. urartu *(6.1%of the total contigs with BLASTX hits) and 4,376 in *T. turgidum *(4.5%, Table 2). Some of these contigs are likely to be the result of transcripts artificially fused during assembly.

As a complementary method to identify and characterize artificially fused contigs(in both orientations) we performed two consecutive runs of findorf. After the initial ORF prediction we masked the predicted coding region and ran a second round of findorf to identify contigs that include more than one predicted protein. We manually annotated 22 putative fusions (see Additional file 2, Table S4) to characterize their origin and evaluate the predictive value of our strategy. Only six contigs (27.3%) included ORFs that mapped to different *T. aestivum *genomic contigs[32].For three of them, we were able to identify a common microsatellite in the UTR, a shared inverted region in the UTR, and a common conserved domain as the probable sources of the incorrect fusions (Additional file 2, Table S4). Among the contigs including two ORFs that were mapped to the same genomic contig, five (22.7%) were fused due to overlapping 3' UTR regions in opposite DNA strands (adjacent genes with opposite orientations). Of the remaining 11 contigs (50%),the inconsistent ORF prediction was associated with the presence of repetitive regions (three cases), pseudogenes and very short predicted secondary ORFs (five cases), and adjacent ORFs that were incorrectly predicted as separate proteins andrepresentedtrue biological gene fusions (three cases) (Additional file 2 Table S4).

To eliminate incorrectly predicted artificially fused contigs we added additional filters to the prediction of secondary ORFs including: (1) elimination of short predicted ORFs (<30 amino acids);(2) elimination of ORFs predicted as pseudogenes; and (3) elimination of ORFs predicted in regions with significant similarity to repetitive elements (TREP database)[49]. After applying these filters, the number of contigs annotated as putative artificially fused transcripts was reduced byhalf (Table 2). Combining both methods (and excluding overlapping contigs), we estimated a total of 4,866 (8.2%) and 6,409 (6.7%) putative artificially fused transcripts for *T. urartu *and *T. turgidum*, respectively (Table 2). Predicted fused transcripts are marked either as 'inconsistent_strand' in the comments field or 'predicted_iter2_orf' in the source fieldof the GTF annotation files (Supplemental datasets 4 and 5[36]), depending on which of the two methods was used to identify the putative fusion.

In summary, after addition of the secondary ORF predictionsidentified in the artificially fused contigs and the exclusion of predicted pseudogenes,the final transcriptome datasets comprised 37,806 ORFs in *T. urartu *(Supplemental dataset 4[36]) and 66,633 ORFs in *T. turgidum *(Supplemental dataset 5[36]). The predicted proteins from these ORF are deposited in Supplemental dataset 6 (*T. urartu*)and Supplemental dataset 7 (*T. turgidum*)[36].

### Gene structure

A BLASTN comparison between our transcriptomes and the available genomic sequences for the Chinese Spring chromosome arms [32] allowed us to simultaneously determine gene structure and chromosome location (Supplemental datasets 13 and 14[36]). A threshold of 99% identity was used to identify the most likely correct homoeolog for each of our predicted ORFs. The analysis of the BLASTN results showed that 46% of the *T. urartu *and 55%of the *T. turgidum *ORFs have ≥99% identity (and ≥65%coverage) to one or more contigs of Chinese Spring (Table 3). These results indicate that roughly half of our ORFs are represented by the corresponding homoeologous genome in the current genomic assemblies of the wheat chromosome arms, with 40% in *T. urartu *and 50% in *T. turgidum *being full length (>95% coverage). Significant alignments with the other homoeolog (94%≤ Id < 99%, >65% coverage) were identified for another 42% and 33% of the *T. urartu *and *T. turgidum *ORFs, respectively.

**Table 3:** Comparison of predicted ORFs (excluding pseudogenes) with *T. aestivum *genomic DNA contigs

Transcriptome	*T. urartu*	*T. turgidum*
*Putative correct homoeolog (Id ≥ 99% identity)* ≥95% coverage in one CS contig	14,678	32,554
≥95% coverage in more than one CS contig	489	911
≥65% coverage in one or more CS contigs	2,094	3,136
*Putative homoeolog from different genome (94%≤ Id < 99%)* ≥95% coverage in one CS contig	12,239	17,437
≥95% coverage in more than one CS contig	1,146	1,549
≥65% coverage in one or more CS contigs	2,416	3,262
Not aligned		
Id <94% or coverage <65%)	4,549	7,370
Number of query sequences with no significant BLAST hits (e^-10^)	195	414

Total number of query sequences	37,806	66,633

These alignments were used to predict gene structure using the program EXONERATE [50]for all the ORFs. We identified complete gene structures (>95% coverage) for 77.6% of the ORFs and at least partial structures (>65% coverage) for 88.0% of the ORFs (Table 3 weighted averages of the two datasets). The coordinates of the predicted exons are provided in Supplemental Datasets 13 (*T. urartu*) and 14 (*T. turgidum*)[36]. These tables also provide percent identities between the predicted ORFs and the Chinese Spring contigs (Table 3) and can be used to infer homoeologs among the *T. turgidum *ORFs.

### Phasing of merged homoeologs to reconstruct genome-specific sub-assemblies

**Figure 6:**
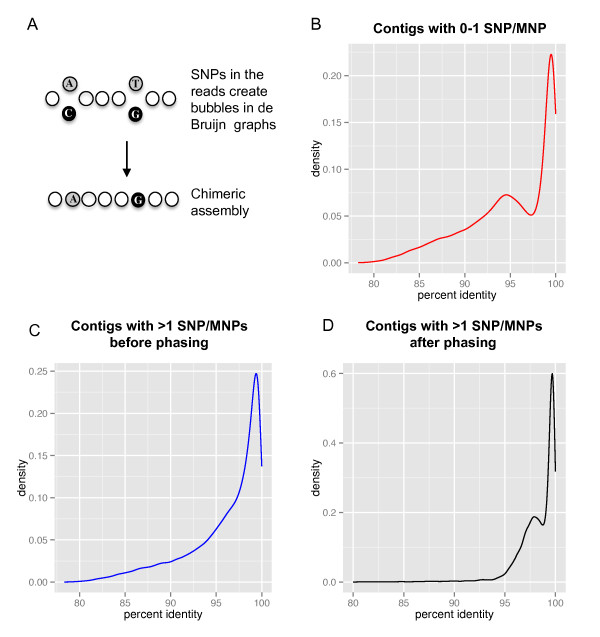
**Identification and phasing of A/B contigs merged during the assembly**. (**A**) Schematic illustration of a contig merged during the assembly. Empty circles represent nucleotides that are common between homoeologs. Grey and black circles correspond to biological polymorphisms between homoeologs. (**B**) Density plots of percent identity between *T. turgidum *and *T. urartu*for contigs with <2 SNPs. The 95% identity peak represents mostly B genome contigs and suggests a relatively good separation of A and B genome contigs in this dataset. (**C**,**D**) Density plots of percent identity between *T. turgidum *and *T. urartu *for contigs with ≥2 SNPs. (C) Distribution before phasing (note the absence of a bimodal distribution) and (**D**) after phasing (bimodal distribution as in B).

Based on previous reports [27], we expected that even using very sensitive assemblers, a significant proportion of the homoeologs would be merged creating A/B chimeric contigs (Figure 6A). Therefore, we exploredpost-assembly approaches to separate merged contigs.We hypothesized that the separation of two homozygous genomes in a self-pollinated- and therefore, highly homozygous -tetraploid species presents similar challenges to the separation of haplotypes in a sexually reproducing diploid organism. The problem of resolving heterozygous haplotypes from next generation sequencing data has recently been tackled in humans [51] using the HapCUT algorithm[52].

To identify polymorphisms inmerged homoeologs we first re-aligned all the *T. turgidum *reads back to the assembled contigsallowing a maximum of 10 mismatches per 2×100bp paired-end read fragment (>95% identity). Using the FreeBayes variant detection program with parameters adjusted for RNA-seq data (see Materials and methods), we identified 1,179,465 polymorphisms, including 958,362 SNPsand 23,424 multi-nucleotide polymorphisms (MNPs) present in 103,304 contigs (Table 4). There were a total of 74,880 contigs (53.4%) that contained >1 SNP/MNP, which were therefore good candidates for polymorphism phasing (Table 4).As expected, this proportion was much lower in *T. urartu*, which showed only 25.4% of the contigs with >1 SNP (21,926/86,247), which indicates that some close paralogs have been merged in the multi-k-mer diploid assembly.

**Table 4:** Polymorphism detection in the tetraploid wheat assembly and polymorphism phasing

*Polymorphisms before phasing*	
Polymorphisms (*n*)	1,179,465
Singlenucleotide polymorphisms (SNP) (*n*)	958,362
Multi-nucleotide polymorphisms (MNP) (*n*)	23,424
Insertions	72,144
Deletions	39,882
Complex^a^	84,457
Other (>2 alleles)^b^	1,089

*Phasing (HapCUT)*	

Contigs with <2 SNP/MNP (*n*)	65,238

Contigs with >1 SNP/MNP (*n*)	74,880
Phased contigs (*n*)	67,169
Phased blocks (*n*)	81,413
Phased SNPs/MNPs (*n*)	864,865
Chimeric reference contigs (*n*)	34,029

*Readphaser*	

Reads filtered due to mapping quality <30 (*n*)	106,003,190
Reads filtered due to indels (*n*)	6,544,331
Reads passed to MIRA (*n*)	256,016,046

^a^Complex: composite insertions and substitution events.

^b^Other: includes cases with >1 alternative allele.

To test if contigs without SNPs/MNPs were already separated into A/B homoeologs, we plotted the percent identity of twocontig groups (those with and those without SNPs/MNPs) to our *T. urartu *transcriptome (Figure 6B). The population of contigs with <2 SNPs/MNPs(65,238 contigs) showed a bimodal distribution in percent identity corresponding to the predicted distributions of A/A and A/B homoeologous alignments. This indicates that a large proportion of contigs without SNPs/MNPs representwell-separated A or B homoeologs.In contrast, the bimodal distribution is not observed in the contigs with >1 SNP/MNP (Figure 6C, before phasing), likely due toA/B chimeras with intermediate identity values.

Using the HapCUT program[52](see Materials and methods), wephased 88% of the SNPs/MNPs detected by FreeBayes in 67,169tetraploid wheat contigs (Table 4), referred to hereafter as reference contigs.Ideally, each reference contig should be phased into two continuous contigs representing the two homoeologs/paralogs. However, when long stretches without SNPs are present or SNPs cannot be consistently phased, the contig is partitioned into blocks that must be phased independently. In our dataset, roughly 20% of the contigs were partitioned into more than one block (total 81,413 blocks), resulting in an average of 1.2 blocks per contig. Blocks were classified as being either chimeric (alternating A and B phases within the block) or non-chimeric (all SNPs/MNPs in the same phase). Using this criterion, we established that 34,029reference contigs (51% of the phased contigs, or 24% of all contigs)were chimeric and 33,140 (49% of the phased contigs, or 24% of all contigs) were non-chimeric. This last set includes cases where only one homoeolog of a close pair was retained in the assembly after CD-HIT.

Because HapCUT generates tables of phased SNPs but not assembled phased sequenceswe developed a new program readphaser (see Materials and methods, Additional file 7and [53]) that sorts the reads within each block into two phases based on the HapCUT tables. Sorted reads are then re-assembled independently bya combination of MIRA [54]and CAP3 (see Materials and methods). To avoid miss-assembly of recent paralogs, readphaser does not include reads where there is evidence of a third valid haplotype.

The MIRA-CAP3 assembly resulted in 244,165 contigs. Since two phases were submitted for each of the 81,413 phased blocks, this result indicates that our readphaser-MIRA-CAP3 pipeline further partitionedhalf of the submitted blocks (81,399) into >1 contig. To estimate the proportion of sequences from the original contigs that were recovered by the HapCUT-readphaser-MIRA pipeline we aligned the MIRA-CAP3 contigs to the original blocks. These analyses showed that 98% of the original contigs are represented (at least partially) in the current MIRA-CAP3 assembly, but also that the 244,165 MIRA-CAP3 contigs cover only 62% of the original sequences. The sequences not covered include regions of low coverage in the original blocks, long stretches of identical sequences between A and B genomes (Figure 1B), and A/B SNPs that were not used by HapCUT and readphaser due to low mapping quality values (<30). Reduced mapping quality was particularly prevalent in sequences represented by >1 contig with identical regions, such as alternative splicing forms. Because reads derived from these redundant regions can map equally well to multiple locations, their mapping quality is greatly reduced.Roughly 30% of the reads were excluded from the MIRA assembly due to low mapping quality (Table 4), suggesting that there is a delicate balance between the stringency of the mapping quality and the proportion of phased sequences. Possible alternatives to increase coverage of the phased sequences in the future includeadditional reductions in the reference dataset (for example, alternative splicing forms) or the use of different mapping qualitythresholds for phasing.

To evaluate the quality of the phasing results we usedtwo different approaches. First, we compared the HapCUT phased SNPs for our manuallycurated set of 26 homoeologous gene pairs (Supplemental dataset 1[36]) with their known phases. Before phasing, these 26 gene pairs were represented by 33 contigs with 377 SNPs between the A and B genomes. A comparison of the HapCUT tables and the manually curated genes showed that 372of 377SNPs (98.7%) were correctly phased. Therefore, after phasing only 1.3% of the SNPs in 24% of the contigs were still chimeric.Additionally, we compared the phased MIRA assemblies to the *T. urartu *contigs (A genome, one phase). This approach also showed an overall good separation of homoeologs. Before phasing, the distribution of BLASTN similarity values between *T. urartu *and unphased reference contigs showed no evidence of a distinct peak for B genome contigs (Figure 6C). We hypothesize that this is the result of the large proportion of A/B chimeric contigs (51% of the phased contigs with >1 SNP), which generate intermediate similarity values that mask the bimodal distribution. In contrast, the alignments generated after phasing show a clear bimodal distribution (Figure 6D). Together, these data indicate that our post-assembly pipeline significantly reduced the number of chimeric transcripts.

### Conclusions

The comparison between our diploid and tetraploidwheat assemblies showed that merging assemblies across a wide range of k-mersizeshas a positive effect on *de novo *transcriptome assemblies in both diploid and polyploid species, but has a larger positive effect on the latter. We speculate that this is related to the heterogeneity in the distribution of SNPs between homoeologs in the different gene classes, which favors full-length assemblies of different genes at different k-mersizes. However, multiple k-mer assemblies also lead to increasesin sequence redundancy, which require post-assembly processing. This is especially challenging in polyploid species where different chimeric contigs can be assembled at different k-mersizes. We showed that a CD-HIT merge using a 95% identitythreshold, which in wheat includes approximately 95% of the homoeologous regions, resulted in a good balance between assemblyquality and reduced redundancy.

A critical step in a polyploid transcriptome assembly is the separation of homologs. The approach followed by Schreiber *et al*. (2012), that implemented a computationally-intensive two-stage assembly using the stringent MIRA assembler in the last step, reduced the proportion of chimeric contigs to 18% and represented a step in the right direction[27]. Thepost-assembly read phasing pipeline presented in this study represents an advance over current methodsto solve the problem of assembling closely related sequences without generating chimeras.Since this post-assembly pipeline is not dependent on resources specific to wheat, itcouldalso be applied to help resolve similar challenges in assembling transcriptomes ofother homozygous tetraploid species. The only parameter that needs to be adjusted to the level of divergence between the targeted genomes is the maximum number of mismatches allowed in the mapping of the reads back to the contigs for homoeolog SNP discovery.

This specialized bioinformatics pipeline was developed with the main objective to generate a high-quality annotated tetraploid wheat transcriptome. However, some of the new modulesfrom our pipeline, such as readphaser, willlikelyfacilitate development of more general strategiesfor assembling transcriptomes of other tetraploid species.

Comparison of tetraploid wheat contigs with and without phasing indicates that the initial assembly separated well relatively distant homologs (average 95% identity, see Figure 6B) but failed to distinguish between more closely related sequences (average 97.5% identity, Figure 6D). A corollary of this interpretation is that only recently originated polyploid species may require phasing for a correct separation of homoeologs.

Our transcriptome annotation effortsyielded a valuable dataset of coding sequences and proteins in wheat that greatly enrichesthe currently sparse wheat proteomic dataset. These integrated datasets are expected to provide valuable references for RNA-seq and proteomics experiments in wheat.We are using this information to develop a gene capture platform for wheat, which is being used in our laboratories to sequence the exome of tetraploid and hexaploid wheat TILLING populations to identify mutations[55].The predicted tetraploid wheat proteome and gene models generated in this study provide a valuable tool for the wheat research community and for those interested in comparative genomic studies including wheat.

## Materials and methods

### Plant growth conditions and sample collection

The diploid wheat *T. urartu*accession G1812 was selected for this study due to itsclose relationship to the A genome of hexaploid wheat, availability of aBAC library[56] and ongoing genome sequencing project[57]. The tetraploid wheat*T.turgidum *Kronos, a modern durum wheat cultivar with high yield potential and excellent pasta quality, was selected based on the availability of mutant TILLING population [55] and the existence of a genome project at Cold Spring Harbor Laboratory[58].

Wheat grain was surface-sterilized in 10% bleach and incubated at 4°C for 2 days before germination. Young root and shoot tissues were collected 2 to 3 weeks after germination. For Kronos only, spike tissue was collected from mature plants at the booting stage and grain tissue was collected 20 days post anthesis.Sampleswere immediately frozen in liquid nitrogenand stored at -80°C.

### Benchmark gene sets

To test the quality of our assembly pipeline, we used two wheat benchmark sets. The first set consisted of 13,472 full-length non-redundant *T. aestivum *cDNA clones from the *Triticeae*Full-Length CDS DataBase sequenced by traditional Sanger technology [35]. The second dataset consisted of 52wheat genes (26 homoeolog pairs) previously assigned to either the A or B genomes and annotated for gene structure in our laboratory (Supplemental dataset1[36]).

### Library construction and sequencing

Total RNA was extracted using Spectrum^™ ^Plant Total RNA Kit (Sigma), from which mRNA was purified using the Dynabeads mRNA Purification kit (Illumina) and transcribed to cDNA using SuperScript II Reverse transcriptase (Invitrogen) and DNA Polymerase I (Promega). cDNA was purified using the PCR purification kit (Qiagen) and quality was assessed on the Bioanalyzer machine. The cDNAs were fragmented in a Covaris machine (10% duty cycle, Intensity: 4, Cycles per burst: 200, 80 s), treated with end-repair enzymes: T4 DNA polymerase (Invitrogen), Klenow DNA Polymerase (Invitrogen), and T4 Polynucleotide kinase (Invitrogen), and adenylated with Klenow exo (3' to 5' exo minus) (Invitrogen). Illumina PE adapters (Illumina Multiplexing kit, PE-400-1001) were ligated using the Quick Ligation kit (New England Biolabs) and purified with the minElute kit (Qiagen). DNA fragments were separated on a 2% agarose TAE gel; fragment with average sizes of 300, 400, 500, and 600 bp were extracted using Gel Excision tips (Gel Company) and purified using the Gel Extraction kit (Qiagen). Libraries were multiplexed according to the instructions in the Illumina Multiplexing kit (Illumina) with 12 cycles of PCR amplification. Final libraries were purified using Ampure beads in a 1:1 sample volume to bead volume ratio (Ampure). The quality of the libraries was assessed on the Bioanalyzer machine using High Sensitivity DNA kit reagents (Agilent).

Library normalization was performed using double stranded DNA nuclease (Evrogen) as published before[59].Four 300-bp libraries from roots, shoots, spike and grain were pooled for normalization. A total of 250 ng of DNA was allowed to hybridize for 5 h at 68°C in either NaCl or TMAC buffer, equilibrated for 10 min at 68°C in DSN buffer, and digested with 1 μL of DSN enzyme for 25 min at 68°C. A 'no DSN enzyme' control was processed simultaneously to access the normalization efficiency. All samples were re-amplified with 10 to 12 PCR cycles.

All libraries were sequenced using the 100 bp paired-end protocol on four lanes of Illumina HiSeq2000 machines at the University of California Davis (UCD) Genome Center. Base quality calls and demultiplexing was done with the CASAVA 1.8.0 pipeline (Illumina).

### Transcriptome assembly

Overall read quality was assessed using the R package qrqc[60]. Illumina adapter sequences were trimmed with the program Scythe v. 0.981[61](-p 0.2--n 3) and poor quality bases were trimmed with Sickle v. 1.2[62] (-q = 20). Reads arising from common contaminants, including *Homo sapiens *and *Escherichia coli *DNA, wheat mitochondrial and chloroplast sequences as well as wheat rRNA (Additional file 2, Table S3) were identified using BLAT v.34 [63] with the default parameters and then removed.

Artificial sample variation (differences in gene coverage in RNA-seq) and k-mersequencesincluding sequencing errors were removed prior to the assembly using a digital normalization algorithm [33](normalize-by-median.py -C 20 -k 20 -N 4 -x 2e9). A previously constructed wheat RNA-seq library (SRA ERX022241)[34] was used to assess and compare the quality of the assembly before and after normalization as well as to determine optimal parameters for the assembly.

Within each species, combined reads from the different libraries were assembled with CLC Genomics Workbench v. 5.5*de novo *assembly algorithm. Initially, we tested several *de novo *assembly algorithms including Trinity and Oases, and we chose to use CLC due to its performance on the benchmark full-length wheat cDNA datasets and overall assembly parameters. Paired-end distances were specified for each library based on preliminary mapping experiments against benchmark full-length wheat cDNA sequences. Ten individual assemblies were constructed at variable k-mers (word size of 21, 25, 31, 35, 41, 45, 51, 55, 61, 63). A word size of 64 is the maximum permitted when using CLC version 5.5. Other parameters included: bubble size = 400, read mapping = global, and 95% similarity which were chosen based on optimizations using a small read set and the 13,472 full-length wheat cDNA benchmark data.

The individual k-mer assemblies were concatenated and redundancy was reduced using CD-HIT v.4.5.4 [64]. Contig merging was carried out at 95% identity level for *T. turgidum *(cd-hit-est -r 1 -c 0.95 -n 8 -T 0 -gap -2) and 99% identity level for *T.urartu *(cd-hit-est -r 1 -c 0.99-n 8 -T 0 -gap -2).

To reconstruct genes partially assembled at different k-mer lengths, we implemented a protein-guided assembly approach, blast2cap3[39](Additional file 5). Contigs were first clustered based on a common top BLASTX[65] hit (E-value cutoff 1e^-3^)against*T. aestivum*, *Hordeum vulgare*, *Brachypodium distachyon*, *Oryza sativa*, *Sorghum bicolor*, *Zea mays*,and *Arabidopsis thaliana *protein datasets (Additional file 2, Table S3).Each contig cluster sharing a common protein hit was passed to the overlap-based assembly program CAP3 [40](cap3 -p 99 -k 0 -o 100).

To identify contaminating sequences from non-wheat organisms (for example, plant epiphytes and pathogens), we used the following taxonomy-based pipeline at the post-assembly stage. First, all contigs were passed through BLASTX against the NCBI non-redundant (nr) database, retaining the top 10 hits using an E-value cutoff of 1e^-10^. The kingdom-level taxonomy of all hits was retrieved from NCBI's taxonomy data structure using an adapted publicly-available Bioperl script (bp_classify_by_kingdom.pl[66]). Sequences with all top 10hits that matched non-plant organisms were considered likely contaminants and were removed from the assembly using custom Perl scripts.

### ORF prediction and functional annotation

We developed the program findorf to predict ORFs and pseudogenes(Additional file 6) [41].Findorf relies upon BLASTX alignments against protein databasesand includes subcommands: 'findorf join' and 'findorf predict'.The results from BLASTX searches (E-value cutoff 1e^-3^) against plant databases (Additional file 2, Table S3)and HMMER3.0 scans of all contigs translated in all six open reading framesagainst Pfam-A (hmmscan e-value 1e^-3^--domE 1 -noali) were passed to 'findorf join' (--domain-hits) and ORFs were predicted using 'findorf predict' (--evalue 1e^-5 ^--verbose --use-pfam).

Findorf uses a conservative approach to identify translation startsites (TSS)and if an additional methionine exists 5' of the predicted TSS, the information is provided in the GTF files (Supplemental datasets 4 and 5[36]). In cases where HSPs disagree on frame across a majority of alignments, the contig is annotated as having a majority frameshift and the frame of the 5'-most HSP is used during this initial ORF prediction. In cases when findorf detects significant HSPs in opposite strands, it annotates the contig as 'inconsistent strand' and outputs no ORF.

Functional annotation of predicted proteins was done using Hidden-Markov Model based searches against Pfam-A database[42]implemented in HMMER3.0 [67](hmmscan e-value 1e^-3^--domE 1 --noali). Candidate repetitive elements and transposons were identified based on results from BLASTN and BLASTX searches against the nucleotide and protein *Triticeae *Repeat Sequence Databases (TREP)[49] using an E-value cutoff of 1e^-10^.

### Identification of pseudogenes and codon bias analyses

A contig was identified as a putative pseudogeneby findorf when a significant protein alignment (BLASTX, E-value 1e^-5^) between contig sequence and related protein sequences (Additional file 2, Table S3)was disrupted by a premature stop codon or includeda frameshift mutation.In the first case, a significant HSP overlapping the related protein sequenceby >20 amino acids beyond the predicted premature stop codon was required to annotate the contig sequence as a putative pseudogene. In the second case, a contig was annotated as having a frameshift mutation if the HSPs matched different sections of the same reference proteins in two different frames on the same strand.

To further characterize the predicted pseudogenes, we compared codon usage between samples of 3,000 pseudogenes and 3,000 genes that did not include premature stops or frameshifts. Frequencies of codonswere converted to proportions, and Manhattan distances were calculated among the 6,000 data points. To visualize these results, we used a metric multidimensional scaling approach, implemented inthe R language.

### Identification of artificially fused transcripts (>1 ORF)

During the initial ORF prediction, a strand consistency filter was imposed to identify contigs with any BLASTX hits on opposite DNA strands, which gave an estimate of putative merged transcripts. In addition, we used an iterative ORF prediction to identify presence of secondary ORFs. The first iteration of BLASTX alignments (E-value 1e^-3 ^cutoff) were masked and the masked sequences were run a second time through findorf with the same parameters. After manual inspection of the initial results, we imposed additional filtering criteria to identify artificially fused transcripts, including the exclusion of pseudogenes and repetitive elements as well as very short ORFs (<30 amino acids).

### Predicting gene exons and assigning genes to chromosome arms

A BLASTN search with an E-value cutoff of 1e^-10 ^was performed between our *T. turgidum *transcriptome and the genomic sequences of the individual chromosome arms of Chinese Spring generated by the IWGSC[32]. A Perl script was written to process the BLAST output. A hit - tagged with the name of the chromosome arm - was stored if it shared on average ≥94% across all HSPs and was stored together with other contigs that hit the exact same chromosome arm. If the hits to each arm covered ≥65% of the ORF length and matched one or more Chinese Spring contigs, a gene exon-intron prediction model was created with EXONERATE v.2.2.0[50,68](--model est2genome -ryo).

### Phasing SNPs from different homoeologs

To generate genome-specific assemblies in tetraploid wheat, we first aligned *T. turgidum *reads with the *T. turgidum *reference transcriptome (140,118 contigs) using Novoalign software (v. 2.08.01; -F ILM1.8 -o SAM -o Sync -i PE -r Random -t 300) and insert size range specific to each library (see Table 1).We compared bowtie, bwa, and Novoalign and selected the latter because it maximized our quality control parameters (most reads aligned and most reads aligned in proper pairs). Polymorphisms among the mapped readswere detectedusing the FreeBayes software[69](v.0.9.6; parameters:-p 2 -k --min-alternate-count 2 -p 2 --min-coverage 4 -T 0.05) as it has been shown to perform well on RNA-seq data [70]. Called SNPs and MNPs were phased using the HapCUTv.0.5software[52]with default parameters. All phased SNPs are reported in HapCUT tabular format in Supplemental dataset 11[36].

### Assembling phased reads into homoeolog-specific sequences

To generate homoeolog-specific sub-assemblies we tested three different strategies. First, we tried to use the initial reference contig and replace the phased SNPs. However, the presence of non-phased SNPs due to low mapping quality and indels resulted in residual chimeric sequences. Second we attempted to reconstruct consensus sequences from the phased reads based on mapping positions relative to the reference contig, but the presence of indels between the A and B genomes (particularly in UTR regions) complicated the correct reconstruction of consensus sequences. Finally, we were successful in reconstructing homoeolog-specific sub-assemblies by sorting the reads within each phased SNP block based on the HapCUT output, and *de novo *re-assembling the reads for each block and phase using parallelized runs ofMIRA assembler [54].

To sort the reads by phase we developed the program readphaser (Additional file 7)[53]. Readphaser extracts reads that include haplotype-specific SNPs identified by HapCUT and separates them into two phased sets that are independently passed to MIRA. Readphaser filters reads with low mapping quality (mq <30),optical or PCR duplicates, or containing indels. Reads containing out of phase variants, due to sequencing error, tri-allelic variants, or more than two real phases (for example, recent duplications) were placed into an additional set of reads that were unused during assembly. Since some out of phase variants may be biologically interesting, readphaser outputs an additional file with the inconsistent variants in reads.

Re-assembly of sorted reads was performed using a custom Perl script created to run parallel instances of MIRA v. 3.2.1[54](parameters -job=denovo,est,Solexa, padded option) on multiple cores. CAP3 [40](using default parameters) was then run with the MIRA contigs generated for each phasing block to further extend the assemblies. To evaluate the coverage of the phased contigs assembled by MIRA, we aligned sequences back to their original contig from our reference transcriptome assembly with global-local alignments of both the forward and reverse complement using the function pairwiseAlignment in the Bioconductor package Biostrings[71]. Alignments with scores <10 (gap open penalty = -8, gap extension penalty = -2) were not considered. Assembly coverage was calculated using the coverage function in the Bioconductor package IRanges[72].

## Data access

The data from this study is linked to the BioProject PRJNA191053 established for *T. urartu *and Bioproject PRJNA191054 for *T. turgidum*. Raw data is available at the Short Read Archive (accession numbers: SRR769749, SRR769750, SRR863375, SRR863376, SRR863377,SRR863384, SRR863385, SRR863386, SRR863387, SRR863389, SRR863390, SRR863391, SRR863394). Filtered contigs are available through the TSA archive under accession numbers GAKL00000000 for *T. urartu *and GAKM00000000 for *T. turgidum*. All supplemental datasets can also be accessed atthe Project Website [36]. A public BLAST site is available at the public USDA GrainGenes database[73].

## Abbreviations

BLAST: Basic Local Alignment Search Tool; DSN: DoublestrandDNAnuclease; EMS: Ethyl Methanesulfonate; HMM: Hidden Markov Model; HSP: HighScoringSegmentPair; IWGSC: InternationalWheatGenomeSequencingConsortium; lncRNAs: longnon-proteincodingRNAs; LRR: LeucineRichRepeats; MNP: Multiple Nucleotide Polymorphism; NBS: NucleotideBindingSite; ORF: Open Reading Frame; SNP: Single Nucleotide Polymorphism;SRA: ShortReadArchive; TSA: TranscriptomeShotgunAssembly; TILLING:TargetingInducedLocalLesionsinGenomes;TSS: TranslationStartSite; UTR: UntranslatedRegion.

## Competing interests

The authors declare that they have no competing interests.

## Authors' contributions

KVK: RNA-seq library preparation, bioinformatics pipeline design, transcriptome assembly and annotation,analyses of the disease resistance gene family, SNP detection, and SNP phasing;VB:bioinformatics software development(blast2cap3, findorf, and readphaser), data analysis, and visualization; PB, SA, CU: gene model prediction; EA and SW: MIRA assemblies;SP, FT, MS, JD:curation team (benchmark genes and quality control). IWGSC contributed the unpublished assemblies of the genomic sequences of the Chinese Spring chromosome arms.KVK and JD designed the study and wrote the first draft of the manuscript. All authors participated in data analyses, contributed to writing andcritical evaluation of the manuscript.

## Endnotes

^a ^Originally published as 38% but corrected recently to 28%

## Supplementary Material

Additional file 1 Members of the International Wheat Sequencing Consortium Click here for file

Additional file 2 Supplemental Tables (Tables S1-S4) Click here for file

Additional file 3 Supplemental Figure S1 Click here for file

Additional file 4 Supplemental Figure S2 Click here for file

Additional file 5 blast2cap3 program Click here for file

Additional file 6 findorf program Click here for file

Additional file 7 readphaser program Click here for file
