# Supplementary material for: Separating homeologs by phasing in the tetraploid wheat transcriptome
Source: Genome Biol. 2013 Jun 25;14(6):R66. doi: 10.1186/gb-2013-14-6-r66 (PMC4053977; doi:10.1186/gb-2013-14-6-r66)
Supplement: Additional file 1 — Members of the International Wheat Sequencing Consortium [file gb-2013-14-6-r66-S1.PDF]

IWGSC Survey Sequencing Initiative Leaders
On Behalf of the IWGSC Coordinating Committee

Overall Project Leadership:	Rogers, Jane	IWGSC
	Eversole, Kellye	IWGSC/Eversole Associates
	Feuillet, Catherine	INRA-GDEC
	Mayer, Klaus	MIPS, Helmholtz Center Munich, German research Center for Environment and Health
Sequencing Providers:	Rogers, Jane	The Genome Analyses Centre (TGAC), IWGSC
	Akhunov, Eduard	Kansas State University
	Alberti, Adriana	Genoscope
	Alsheikh, Muath	Graminor
	Batley, Jacqueline	University of Queensland
	Budak, Hikmet	Sabanci University
	Choulet, Frederic	INRA-GDEC
	Edwards, Dave	University of Queensland, ACPFG
	Ferber, Melanie	University of Dundee
	Feuillet, Catherine	INRA-GDEC
	Gullord, Magne	Graminor
	Handa, Hirokazu	National Institute of Agrobiological Sciences
	Matsumoto, Takashi	National Institute of Agrobiological Sciences
	McLay, Kirsten	TGAC
	Olsen, Odd-Arne	Norwegian University for Life Sciences
	Pozniak, Curtis	University of Saskatchewan
	Praud, Sebastien	Biogemma
	Sande, Simen Rod	Norwegian University for Life Sciences
	Salina, Elena	Institute Cytology And Genetics
	Sharpe, Andrew	DNA Technologies Lab, National Research Council Canada
	Singh, Kuldeep	Punjab Agricultural University
	Wincker, Patrick	Genoscope
	Wright, Jon	TGAC
Bioinformatics:	Caccamo, Mario	TGAC
	Mayer, Klaus	MIPS, Helmholtz Center Munich, German research Center for Environment and Health
	Alaux, Michael	INRA-URGI
	Ayling, Sarah	TGAC
	Gonzalez-Ramirez, Ricardo	TGAC
	Pfeifer, Matthias	MIPS, Helmholtz Center Munich, German research Center for Environment and Health
	Spannagl, Manuel	MIPS, Helmholtz Center Munich, German research Center for Environment and Health
	Quesneville, Hadi	INRA-URGI
Cytogenetics Stocks:	Gill, Bikram	Kansas State University
Sorted Chromosome & DNA Providers:	Dolezel, Jaroslav	Institute of Experimental Botany
	Simkova, Hana	Institute of Experimental Botany
	Kubaláková, Marie	Institute of Experimental Botany
