# Supplementary material for: Separating homeologs by phasing in the tetraploid wheat transcriptome
Source: Genome Biol. 2013 Jun 25;14(6):R66. doi: 10.1186/gb-2013-14-6-r66 (PMC4053977; doi:10.1186/gb-2013-14-6-r66)
Supplement: Additional file 2 — Supplemental Tables (Tables S1-S4) [file gb-2013-14-6-r66-S2.PDF]

Supplemental Tables S1 to S4

Table S1. Illumina libraries generated during this study

Cultivar	Library name	Tissue	Fragment size [a] peak ± SD	Number of raw reads	Short read archive id number
T. turgidum					
Kronos	KR300	young roots	178 ± 26	92,142,982	SRR863377
Kronos	KR500	young roots	315 ± 107	24,272,160	SRR863384
Kronos	KS300	young shoots	227 ± 31	83,248,092	SRR863385
Kronos	KS500	young shoots	348 ± 108	20,349,430	SRR863386
Kronos	KSp400	spike	306 ± 54	137,060,606	SRR863387
Kronos	KSp600	spike	427 ± 132	76,779,488	SRR863389
Kronos	KG400	grain	295 ± 74	23,406,716	SRR863390
Kronos	KG600	grain	335 ± 176	9,347,198	SRR863391
Kronos	DSN	normalized	195 ± 26	22,287,092	SRR863394
T. urartu					
G1812	UR400	young root	279 ± 52	91,002,888	SRR769750
G1812	UR600	young root	391 ± 156	34,131,018	SRR769749
G1812	US400	young shoot	322 ± 62	81,090,136	SRR863375
G1812	US600	young shoot	418 ± 131	42,308,754	SRR863376

[a] Empirical insert sizes for the libraries. Initial size distribution was calculated empirically based on mapping reads back to a benchmark set of 13,472 full-length cDNA sequences from the RIKEN Plant Science Center Japan [1]. All libraries were sequenced on Illumina HiSeq 2000, as 100 bp paired-end reads.

Table S2. Assembly statistics across different k-mers for *T. urartu* and *T. turgidum.*

***T. urartu* (diploid)**

k-mer	21	25	31	35	41	45	51	55	61	63	Merged
Number of contigs	39,255	42,479	44,719	45,829	46,178	46,301	45,170	43,940	41,325	40,320	86,247[a]
Min. contig size (bp)	212	350	405	378	373	425	448	477	477	477	212
Max. contig size (bp)	17,959	16,477	16,477	16,477	16,477	16,462	15,317	16,415	16,232	16,232	17,959
Mean contig size (bp)	1,303	1,288	1,302	1,310	1,332	1,336	1,357	1,366	1,380	1,385	1,392
% reads used in the assembly [b]	56	59	63	66	69	71	73	74	74	74	81
% reads mapping in proper pairs	61	61	62	63	63	63	64	64	64	64	66
% benchmark genes assembled full-length in a single contig [c]	28	33	39	43	49	52	56	58	60	60	68

***T. turgidum* (tetraploid)**

k-mer	21	25	31	35	41	45	51	55	61	63	Merged
Number of contigs	71,857	74,422	79,495	82,457	86,564	87,998	89,773	88,960	86,045	84,748	140,118[a]
Min contig size (bp)	314	373	298	411	404	427	451	454	453	452	298
Max contig size (bp)	24,325	19,429	26,226	19,991	20,278	16,976	16,489	14,827	14,827	17,494	26,226
Mean contig size (bp)	1,131	1,133	1,135	1,134	1,129	1,128	1,123	1,117	1,114	1,110	1,268
% reads used in the assembly [b]	52	53	56	58	62	64	68	69	70	70	80
% reads mapping in proper pairs	61	61	62	62	63	63	63	63	63	63	66
% benchmark genes assembled full-length in a single contig [c]	24	25	28	31	36	39	42	44	46	46	68

[a] Numbers after CD-HIT and blast2cap3 redundancy reduction. Before redundancy reduction there were 435,516 contigs in *T. urartu* and 832,319 in *T. turgidum*.

[b] Percent of reads used by an assembly was assessed by mapping the reads back to the assembly (CLC 5.5, 0.95 similarity, global alignment). The numbers of post-processed-reads used as input for the assembly were 47,299,075 for *T. urartu* and 110,715,043 for *T. turgidum*

[c] Full-length assemblies were those with >90% of the query length covered by a single contig at >90% identity. The benchmark genes used in this evaluation included the 13,472 full-length open reading frame wheat sequences derived from Sanger-sequenced cDNAs at RIKEN Plant Science Center Japan [1].

Table S3. Publicly available databases that were used in this study

Database name	Version	Source	Ref.
**Contaminant Removal**			
Homo sapiens	NC_000001-<br>NC_000024	www.ncbi.nlm.nih.gov	[2]
Escherichia coli	NC_012947.1<br>NC_012759.1<br>NC_009800.1<br>NC_010473.1<br>NC_011415.1	www.ncbi.nlm.nih.gov	n/a
T. aestivum mitochondrial genome	NC_007579.1	www.ncbi.nlm.nih.gov	[3]
T. aestivum chloroplast genome	NC_002762.1	www.ncbi.nlm.nih.gov	[4]
Poaceae rDNA	SILVA v.108	www.arb-silva.de	
Nr	Retrieved on 05/28/2012	ftp.ncbi.nih.gov/blast/db/FASTA/nr	n/a
NCBI taxonomy	Retrieved on 10/16/2012	ftp://ftp.ncbi.nih.gov/pub/taxonomy/	n/a
**Blast2cap3, ORF prediction, Functional annotation, R-gene analyses**			
Pfam-A	Retrieved on 11/28/2011	ftp://ftp.sanger.ac.uk/pub/databases/ Pfam	[5]
Oryza sativa	IRGSP-1.0	rapdb.dna.affrc.go.jp	[6, 7]
	Phytozome v8.0	www.phytozome.net	
Sorghum bicolor	Phytozome v8.0	www.phytozome.net	[8]
Brachypodium distachyon	v1.2	brachypodium.org	[9]
Zea maize	Phytozome v8.0	www.phytozome.net	[10]
	ZmGDB	www.plantgdb.org/ZmGDB/	
Arabidopsis thaliana	TAIR10	www.phytozome.net	[11]
Hordeum vulgare	BarleyDB	trifldb.psc.riken.jp	[1]
	HighConf_genes_ MIPS_23Mar12		[12]
	GeneBank	trifldb.psc.riken.jp	[1]
T. aestivum	WheatDB	trifldb.psc.riken.jp	[1]
	GeneBank	trifldb.psc.riken.jp	[1]
**Repeat masking**			
TREP protein	v.10	wheat.pw.usda.gov/ITMI/Repeats/	[13]
TREP nucleotide	v. 10	wheat.pw.usda.gov/ITMI/Repeats/	[13]
**Complementary wheat datasets**			
T. aestivum genome	Survey sequences	www.wheatgenome.org	n/a
T. aestivum transcriptomes	As published	Obtained from authors	[14-16]
T. aestivum full length cDNA	n/a	trifldb.psc.riken.jp	[1]

Table S4. Summary of manually annotated artificially fused transcripts

*T. urartu c*ontig name*	Genomic contig sequence
**ORFs in different chromosome regions**	
tu-k21_contig_7543	No common contig
tu-k21_contig_1110	No common contig
tu-k41_contig_1292	No common contig
tu-k31_contig_12715	No common contig
tu-k63_contig_13695;tu-k35_contig_9428	No common contig
tu-k51_contig_7865;tu-k31_contig_10065	No common contig
**Adjacent ORFs with overlapping 3'UTRs in opposite strands**	
tu-k21_contig_5280;tu-k25_contig_12639;tu-k41_contig_11613	IWGSC_chr3AL_k71_contig_4101607
tu-k21_contig_16064;tu-k31_contig_30814	IWGSC_chr1AL_v2_k71_contig_3891789
tu-k21_contig_1426;tu-k61_contig_725	IWGSC_chr1AL_v2_k71_contig_2770634
tu-k31_contig_293	IWGSC_chr5AL_k95_contig_1594861
tu-k25_contig_9589	IWGSC_chr7BS_k71_contig_3069533
**ORF in repetitive regions**	
tu-k61_contig_30905	IWGSC_chr4AS_V2_k71_contig_5987640 (ORF1~LINE)
tu-k41_contig_20534	IWGSC_chr1AS_k71_contig_3295843 (ORF2~Retro)
tu-k21_contig_27745	ORF1 and ORF2 both ~CACTA DNA transposon Caspar
**Short ORFs and pseudogenes**	
tu-k63_contig_31475	IWGSC_chr5AS_k95_contig_1502365.
tu-k31_contig_34473	IWGSC_chr7AL_k71_contig_4526182
tu-k35_contig_36865 RC	IWGSC_chr2AL_k71_contig_6301223
tu-k55_contig_24028	IWGSC_chr4AL_k71_contig_7160083
tu-k55_contig_17912;tu-k41_contig_20543	ORF1 & ORF2 match wheat EST BJ315986
**ORF in same protein incorrectly predicted as separate ones**	
tu-k51_contig_2137	IWGSC_chr6AL_k71_contig_5754918 & EEE57355.1
tu-k55_contig_8429	IWGSC_7BL_contig_6742371 and AFW60093.1
tu-k61_contig_4567	IWGSC_chr6AL_k71_contig_5787693

* Contig names separated by ";" were merged using the *blast2cap3* program

References:

1. Mochida K, Yoshida T, Sakurai T, Ogihara Y, Shinozaki K: TriFLDB: a database of clustered full-length coding sequences from *Triticeae* with applications to comparative grass genomics. *Plant Physiol* 2009, 150:1135-1146.
2. Lander ES, Linton LM, Birren B, Nusbaum C, Zody MC, Baldwin J, Devon K, Dewar K, Doyle M, FitzHugh W, et al: Initial sequencing and analysis of the human genome. *Nature* 2001, 409:860-921.
3. Ogihara Y, Yamazaki Y, Murai K, Kanno A, Terachi T, Shiina T, Miyashita N, Nasuda S, Nakamura C, Mori N, et al: Structural dynamics of cereal mitochondrial genomes as revealed by complete nucleotide sequencing of the wheat mitochondrial genome. *Nucleic Acids Res* 2005, 33:6235-6250.
4. Ogihara Y, Isono K, Kojima T, Endo A, Hanaoka M, Shiina T, Terachi T, Utsugi S, Murata M, Mori N, et al: Structural features of a wheat plastome as revealed by complete sequencing of chloroplast DNA. *Mol Genet Genomics* 2002, 266:740-746.
5. Finn RD, Mistry J, Tate J, Coggill P, Heger A, Pollington JE, Gavin OL, Gunasekaran P, Ceric G, Forslund K, et al: The Pfam protein families database. *Nucleic Acids Res* 2010, 38:D211-222.
6. Tanaka T, Antonio BA, Kikuchi S, Matsumoto T, Nagamura Y, Numa H, Sakai H, Wu J, Itoh T, Sasaki T, et al: The Rice Annotation Project Database (RAP-DB): 2008 update. *Nucleic Acids Res* 2008, 36:D1028-1033.
7. International Rice Genome Sequencing Project: The map-based sequence of the rice genome. *Nature* 2005, 436:793-800.
8. Paterson AH, Bowers JE, Bruggmann R, Dubchak I, Grimwood J, Gundlach H, Haberer G, Hellsten U, Mitros T, Poliakov A, et al: The *Sorghum bicolor* genome and the diversification of grasses. *Nature* 2009, 457:551-556.
9. The International *Brachypodium* Initiative: Genome sequencing and analysis of the model grass *Brachypodium distachyon*. *Nature* 2010, 463:763-768.
10. Schnable PS, Ware D, Fulton RS, Stein JC, Wei F, Pasternak S, Liang C, Zhang J, Fulton L, Graves TA, et al: The B73 maize genome: complexity, diversity, and dynamics. *Science* 2009, 326:1112-1115.
11. Lamesch P, Berardini TZ, Li D, Swarbreck D, Wilks C, Sasidharan R, Muller R, Dreher K, Alexander DL, Garcia-Hernandez M, et al: The Arabidopsis Information Resource (TAIR): improved gene annotation and new tools. *Nucleic Acids Res* 2011, 40:D1202-1210.
12. Mayer KF, Waugh R, Brown JW, Schulman A, Langridge P, Platzer M, Fincher GB, Muehlbauer GJ, Sato K, Close TJ, et al: A physical, genetic and functional sequence assembly of the barley genome. *Nature* 2012, 491:711-716.
13. Wicker T, Matthews DE, Keller B: TREP: a database for *Triticeae* repetitive elements. *TRENDS in Plant Science* 2002, 7:561-562.
14. Schreiber AW, Hayden MJ, Forrest KL, Kong SL, Langridge P, Baumann U: Transcriptome-scale homoeolog-specific transcript assemblies of bread wheat. *BMC Genomics* 2012, 13:492.
15. Brenchley R, Spannagl M, Pfeifer M, Barker GL, D'Amore R, Allen AM, McKenzie N, Kramer M, Kerhornou A, Bolser D, et al: Analysis of the bread

wheat genome using whole-genome shotgun sequencing. *Nature* 2012, 491:705-710.

16. Cantu D, Pearce SP, Distelfeld A, Christiansen MW, Uauy C, Akhunov E, Fahima T, Dubcovsky J: Effect of the down-regulation of the high *Grain Protein Content* (*GPC*) genes on the wheat transcriptome during monocarpic senescence. *BMC Genomics* 2011, 12:492.
