# Supplementary material for: Separating homeologs by phasing in the tetraploid wheat transcriptome
Source: Genome Biol. 2013 Jun 25;14(6):R66. doi: 10.1186/gb-2013-14-6-r66 (PMC4053977; doi:10.1186/gb-2013-14-6-r66)
Supplement: Additional file 3 — Supplemental Figure S1 [file gb-2013-14-6-r66-S3.PDF]

Figure S1.

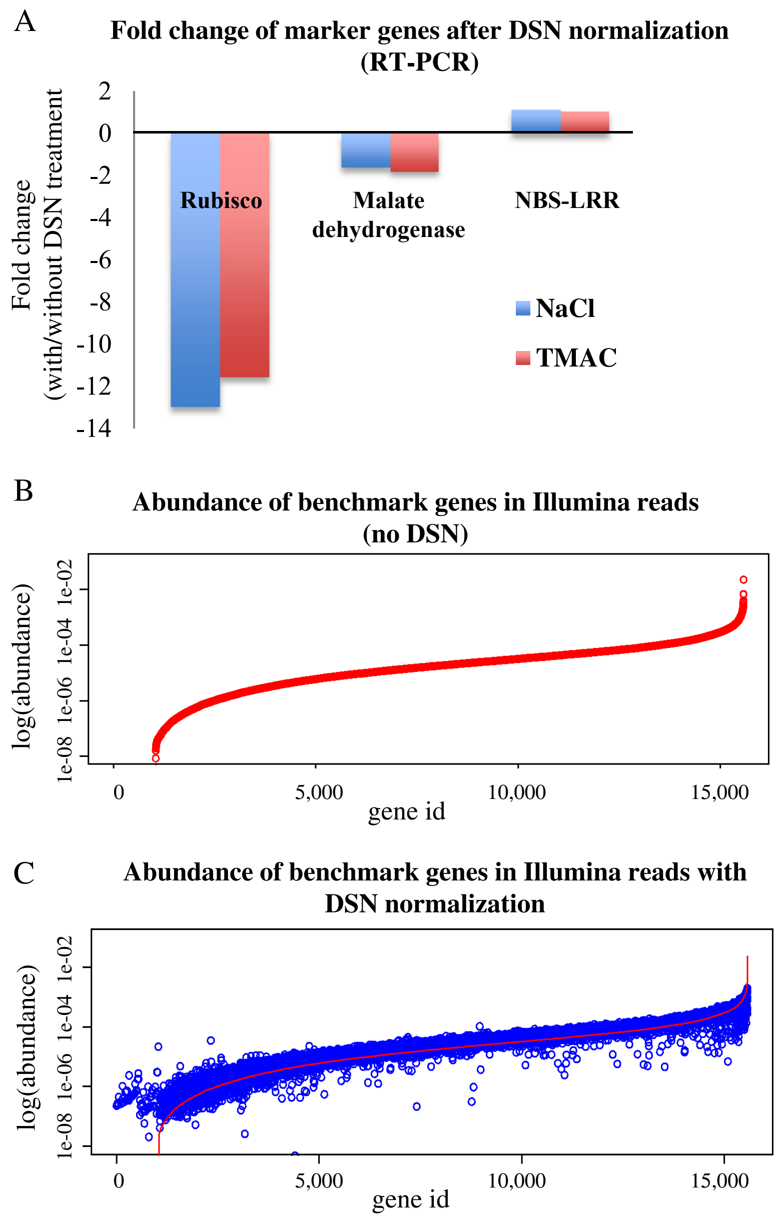

Figure S1. The Double Stranded Nuclease (DSN) normalization enriches low expression transcripts
(A) Fold change in abundance of three marker genes in Illumina libraries with and without DSN treatment. Note the reduction in abundance of highly expressed genes (Rubisco and Malate dehydrogenase) and the increase in the low abundance gene (NBS-LRR). Two hybridization buffers, NaCl (blue) and TMAC (red) were compared. Fold change was calculated using two biological replicates per hybridization buffer and the ratio between $2^{(C_T\text{Gene}-C_T\text{Actin})}$ values in DSN normalized and control libraries. (B) Relative abundance (y-axis) for each benchmark transcript (x-axis) in the sequenced reads of the control Illumina library not treated with DSN. Transcripts are sorted from most abundant (on the right) to least abundant (on the left). Reads were aligned using CLC genomics (99% identity, global alignment) and relative abundance values were calculated using the RSEM package. (C) Relative abundance of the same transcripts with DSN treatment. A red line overlaid on the graph shows the abundance values corresponding to no DSN treatment. Note the decrease in the most abundant genes (on the right) and the increase in the least abundant (on the left).
