# Supplementary material for: Separating homeologs by phasing in the tetraploid wheat transcriptome
Source: Genome Biol. 2013 Jun 25;14(6):R66. doi: 10.1186/gb-2013-14-6-r66 (PMC4053977; doi:10.1186/gb-2013-14-6-r66)
Supplement: Additional file 4 — Supplemental Figure S2 [file gb-2013-14-6-r66-S4.PDF]

Figure S2.

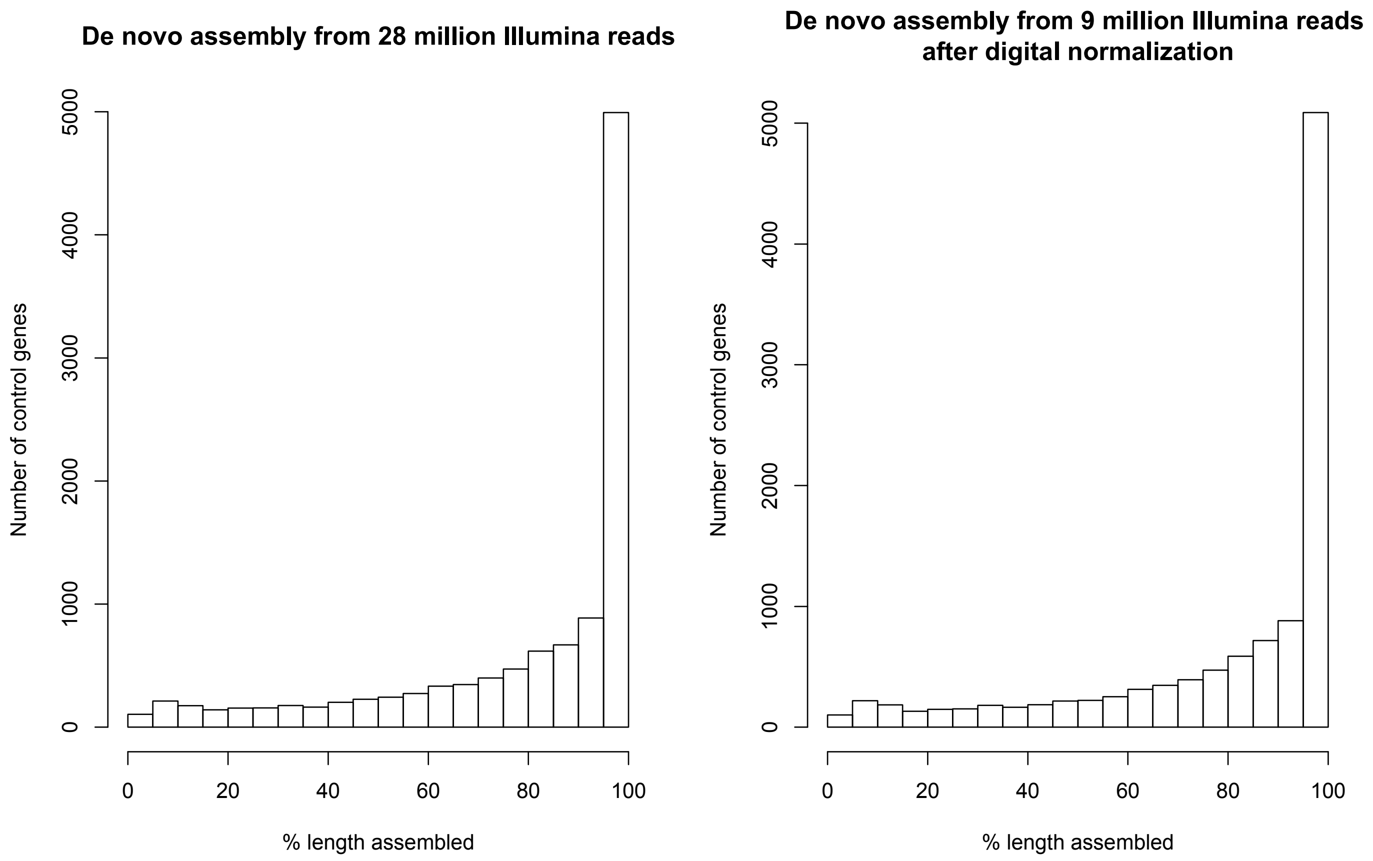

Figure S2. Digital normalization of reads with khmer does not affect assembly quality
The effect of digital normalization with khmer was tested on a subset of reads. Reads with and without normalization were passed through a multiple k-mer assembly pipeline. Benchmark cDNA sequences were aligned to both assemblies using BLASTN (E-value cutoff $1e^{-20}$ and >90% identity) and distribution of length coverage was plotted with assembly completeness of benchmark sequences (% length) on the x-axis and number of assembled sequences on the y-axis.
